# Genetic mosaic dissection of candidate genes in mice using mosaic analysis with double markers

**DOI:** 10.1016/j.xpro.2021.100939

**Published:** 2021-11-10

**Authors:** Nicole Amberg, Simon Hippenmeyer

**Affiliations:** 1 Institute of Science and Technology Austria, Am Campus 1, 3400 Klosterneuburg, Austria

**Keywords:** Single Cell, Developmental biology, Genetics, Microscopy, Model Organisms, Molecular Biology, Neuroscience, Stem Cells, Tissue Engineering

## Abstract

Mosaic analysis with double markers (MADM) technology enables the generation of genetic mosaic tissue in mice. MADM enables concomitant fluorescent cell labeling and introduction of a mutation of a gene of interest with single-cell resolution. This protocol highlights major steps for the generation of genetic mosaic tissue and the isolation and processing of respective tissues for downstream histological analysis.

For complete details on the use and execution of this protocol, please refer to Contreras et al. (2021).

## Highlights

• Generation of genetic mosaic mice using mosaic analysis with double markers (MADM)
• Tissue harvesting from experimental MADM mice
• Processing and imaging of MADM-labeled tissue
• Distinct analyses to assess cell-autonomous gene function in MADM mice

## Graphical abstract

**Figure:**
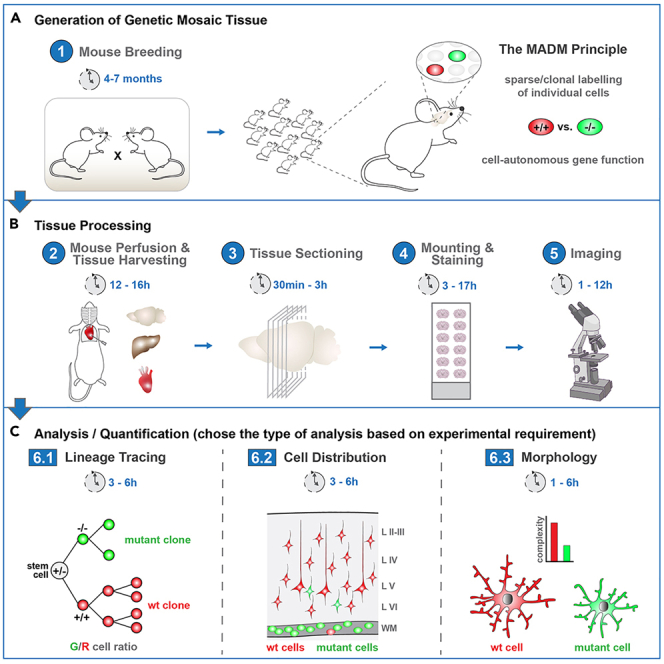

In this protocol we established an experimental pipeline for the generation of experimental MADM mice; and isolation of MADM-labeled tissue for downstream histological analysis.

## Before you begin

### Background

This protocol describes the generation of genetic mosaic mice using mosaic analysis with double markers (MADM) (Zong et al., 2005; Contreras et al., 2021). A genetic mosaic individual consists of cells of distinct genotypes, for example harboring homozygous mutant cells next to wild-type cells. This phenomenon occurs naturally and is widespread across multicellular organisms. The advantages of using genetic mosaic animals for experimental studies are manifold: The controlled induction of genetic mosaicism in experimental animals allows to alter gene function at high spatiotemporal resolution and has led to many fundamental discoveries of gene function and cellular mechanisms in diverse biological contexts. Genetic mosaicism can be induced sparsely, so that individual mutant cells will be surrounded by unperturbed cells. Thus, mosaicism enables the study of cell-autonomous gene function at single cell resolution. Individual mutant cells can be studied in many ways, for example in lineage tracing, cell competition assays, morphological analysis and disease modelling (Hippenmeyer et al., 2010; Gao et al., 2014; Joo et al., 2014; Beattie et al., 2017; Tian et al., 2020; Contreras et al., 2021).

In this protocol, we present an experimental pipeline for the generation of MADM-labeled mosaic tissues and highlight a variety of downstream analysis applications. Our protocol is based on the use of distinct Cre drivers in combination with MADM cassettes targeted to all 19 mouse autosomes (Contreras et al., 2021).

**Figure 1:**
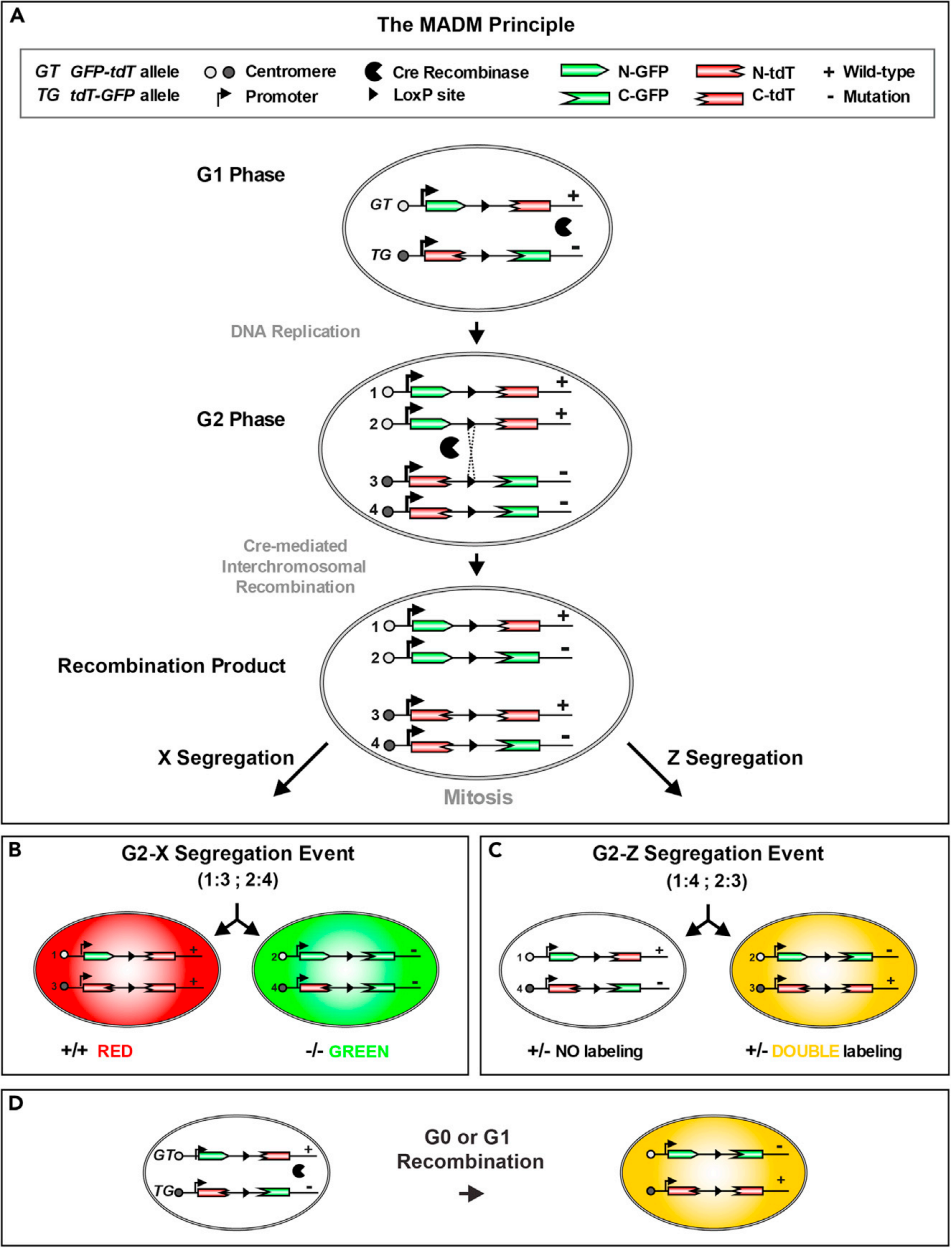
The MADM principle (A) For MADM, two reciprocally chimeric marker genes are targeted to identical loci on homologous chromosomes. The chimeric marker genes (*GT* and *TG* alleles) consist of partial coding sequences for green (eGFP[G]) and red (tdT[T]) fluorescent proteins separated by an intron containing the *loxP* site. Following Cre recombinase-mediated interchromosomal recombination during mitosis, functional green and red fluorescent proteins are reconstituted. (B) G2-X segregation of the recombinant chromosomes results in two daughter cells each expressing one of the two fluorescent proteins (recombination in G2 of the cell cycle followed by X segregation). Introduction of a mutation distal to one MADM cassette (here the TG cassette) allows the generation of genetic mosaics with single cell resolution, with wild-type daughter cells labeled in red and homozygous mutant sibling cells labelled in green in an unlabeled heterozygous environment. (C) G2-Z segregation results in two daughter cells with no genotype alteration. One cell shows double labeling (= yellow), while the other daughter cell is unlabeled. (D) G1 and G_0_ events also result in yellow cell labeling without change in genotype. Reproduced and adapted with permission from (Contreras et al., 2021).

Mosaic analysis with double markers (MADM) is a genetic technology that allows concomitant fluorescent labeling and introduction of a homozygous mutation of choice with unprecedented single cell resolution in a defined genetic lineage (Zong et al., 2005; Contreras et al., 2021). MADM is based on the use of split marker genes that consist of two reciprocal cassettes of partial coding sequences for green fluorescent protein (GFP) and tandem dimer Tomato (TdT), interspersed by *loxP* sites (Figure 1A). The two reciprocal MADM cassettes need to be maintained in two different mouse lines, the GT (N-GFP/C-TdT) line and the TG (N-TdT/C-GFP) line (Zong et al., 2005; Hippenmeyer et al., 2010).

In order to generate genetic mosaic tissue, a modified gene of interest needs to be meiotically recombined with one of the MADM chromosomes (here shown as coupled to the TG line, resulting in an animal being heterozygous for the gene modification of interest). Additionally, one of the lines needs to carry a Cre driver of interest for the generation of experimental animals. Cells with MADM-induced mosaicism are generated by Cre/*loxP*-dependent mitotic interchromosomal recombination during the G2 phase in dividing progenitor/stem cells followed by X-segregation (that is the transmission of the two recombinant chromosomes to distinct daughter cells, resulting in individual green (GFP^+^) mutant and red (tdT^+^) wild-type cells) (Figures 1A and 1B). Recombination in the G2 phase of the cell cycle with segregation of both recombinant chromosomes to the same daughter cell (Z-segregation) does not alter the genotype and results in one yellow GFP^+^/tdT^+^ heterozygous cell which serves as control. At the same time, Z-Segregation results in transmission of the non-recombined chromosomes to the other sibling daughter cell and thus gives rise to an unlabeled cell (unchanged genotype, heterozygous) (Figure 1C). G1 and/or postmitotic G_0_ events also result in yellow cells (Figure 1D).

Importantly, the existence of sparse green mutant, red wild-type and yellow heterozygous cells in an otherwise unlabeled heterozygous environment not only allows to study gene function, but also to perform gene dosage analysis at the individual cell level within the same tissue, in a cell-type-specific manner.

Here, we demonstrate the coupling of the mutant allele of interest with the TG line, which upon successful Cre-mediated recombination will result in genetic mosaic tissue consisting of sparse genetically modified cells labeled in green, while corresponding wild-type cells will be labeled in red and all other cells (yellow and unlabeled) will be heterozygous (Figure 1).

For more details on the MADM technology, please refer to Zong et al. (2005), Beattie et al. (2020), Laukoter et al. (2020a), and Contreras et al. (2021).

For the successful generation of experimental MADM mice, a number of considerations have to be taken into account before starting with breeding (also see troubleshooting problem 1):

1. Institutional and governmental permission and oversight information for the animal study should be obtained. In this study, experimental procedures were discussed and approved by the institutional ethics and animal welfare committees at IST Austria in accordance with good scientific practice guidelines and national legislation (license number: BMWF-66.018/0007-II/3b/2012 and BMWFW-66.018/0006-WF/V/3b/2017).
2. For MADM to work, the presence of a Cre recombinase, active in stem or progenitor cells of your tissue of interest, is required. In other words, any Cre-driver can be used, provided Cre is expressed in dividing stem and progenitor cells, to target cell types and lineages of interest.
3. MADM can be employed for both population and clonal analysis. Depending on the aim of your experiments, you are required to choose a Cre driver enabling either population analysis (constitutively active Cre) or clonal analysis (e.g., tamoxifen (TM)-inducible CreER driver).

Population analysis allows the analysis of a high number of single, sparsely labeled cells in one organ to obtain a highly quantitative read-out. However, constitutively active Cre will result in MADM events at any time point during the entire time window of Cre activity (e.g., *Emx1*-Cre activity starts at ∼E9.5 and is detectable up to early postnatal age). Given this relatively long activity window, the detection of labeled cells does not provide information about the spatiotemporal origin of these cells or the stem cell division mode.

In order to get to spatiotemporally controlled induction of MADM events clonal analysis, employing a TM-inducible CreER driver, can be used. Injection of one single low dose of TM allows the induction of G2-X MADM events only at the injection time point, thus generating high spatiotemporal resolution and conserving information about stem cell division modes. For more detailed information about the usage of the MADM system for clonal analysis, please refer to (Beattie et al., 2020).

4. Make use of meiotic recombination in the germline to genetically link a mutant allele of your gene of interest to the corresponding MADM chromosome (Hippenmeyer et al., 2010; Laukoter et al., 2020b) (Figure 1). The probability for meiotic recombination that results in the linkage of the mutant allele with the MADM cassette can be estimated: By determining the genetic distance (centi-Morgan [cM]) between the gene locus of interest and the MADM cassette on a particular chromosome by using for example Mouse Genome Informatics (MGI) database (www.informatics.jax.org) (Figure 2A).

**Figure 2:**
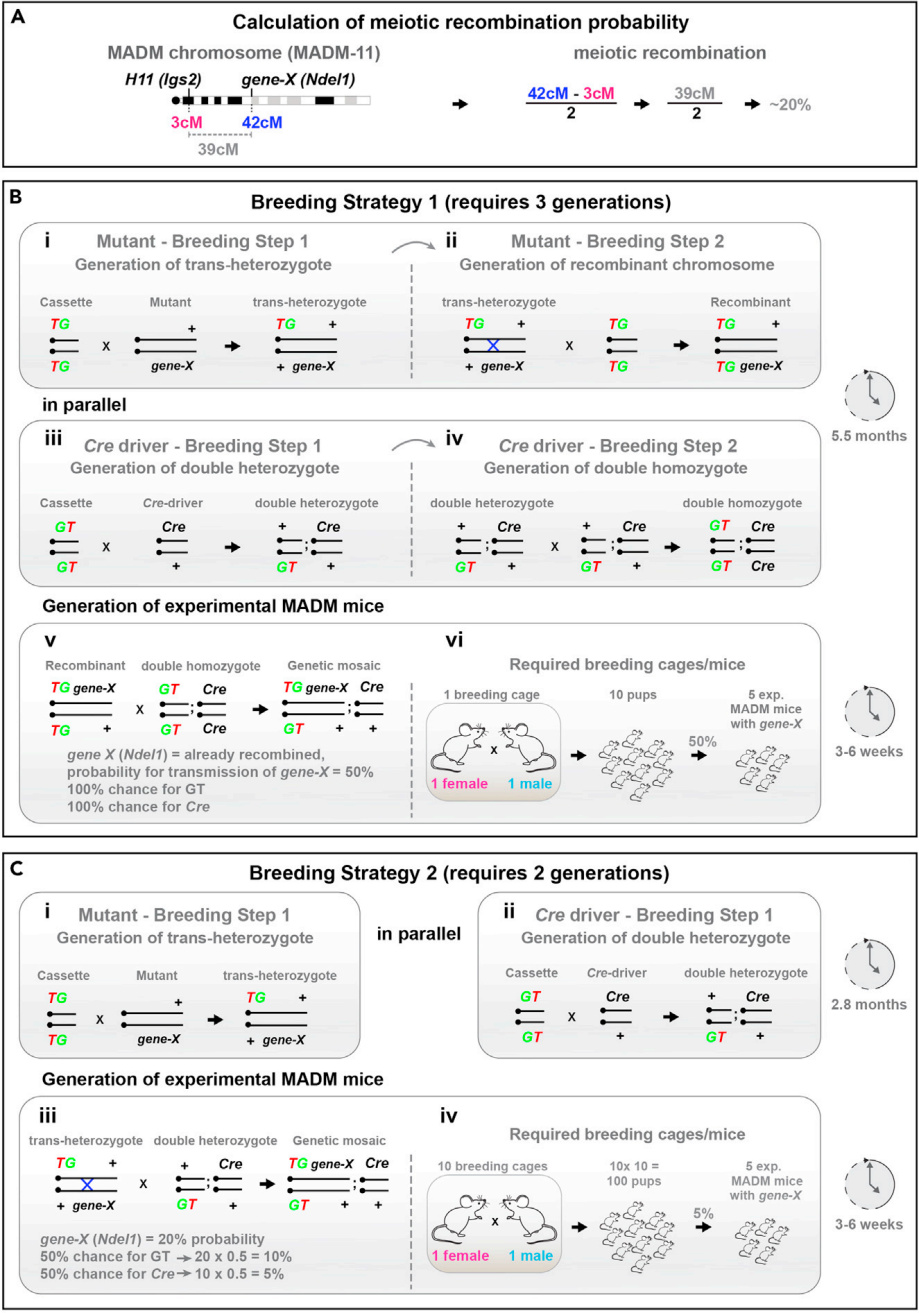
Calculation of meiotic recombination frequency and breeding schemes for MADM mice (A) Calculation of meiotic recombination frequency to genetically link the gene of interest (here *Ndel1*) to the corresponding MADM chromosome (here MADM-11). (B) Breeding strategy 1 for maximal output efficiency in the generation of experimental MADM mice. (i) In the first breeding step, the mouse line carrying the mutation of interest is bred to the TG cassette of the corresponding MADM chromosome. (ii) In a second step, MADM cassettes are homozygosed by crossing the trans-heterozygote offspring from (i) with animals homozygous for the TG cassette. Thereby, the mutation of *gene-X* is genetically linked (through meiotic recombination) to the MADM TG cassette on the corresponding MADM chromosome. (iii) In parallel to steps (i) and (ii), the first breeding step for the Cre driver is performed by crossing the Cre line of choice with the GT line. (iv) In a second step, MADM cassettes are homozygosed by performing an intercross of the double heterozygous offspring from (iii). (v) Mating setup resulting in experimental MADM mice with mutant cells labelled in green and wild-type cells labelled in red. (vi) Summary of the required breeding cages to obtain 5 experimental MADM mice. The breeding strategy allows to obtain 50% of genetic mosaic MADM animals from one experimental mating. (C) Breeding strategy 2 for maximal temporal efficiency in the generation of experimental MADM mice. (i) In the first breeding step, the mutation of *gene-X* is genetically linked to the MADM TG cassette on the corresponding MADM chromosome. (ii) In parallel, the Cre driver is bred to the GT line. All mice of the F1 generation will be heterozygous for MADM cassettes, *gene-X* and *Cre*, respectively. (iii) The breeding step rendering the MADM cassettes homozygous is skipped and F1 mice are used directly to set up the mating resulting in experimental MADM animals with mutant cells labelled in green and wild-type cells labelled in red. (iv) Summary of the required breeding cages to obtain 5 experimental MADM mice. The strategy allows to obtain mosaic animals at a frequency of ∼5% from ∼10 experimental matings.
5. Choose MADM chromosomes according to the chromosomal location of your gene of interest. For example, if your gene of interest is located on chromosome 7, you need to recombine it with a MADM cassette localized on chr.7 (MADM-7). If your gene of interest is located on chromosome 11 (as shown in our example in Figure 2), you need to recombine it with a MADM cassette localized on chr.11 (MADM-11). We have reported distinct MADM recombination frequencies for different MADM chromosomes. Consider the frequency when evaluating your results, particularly when frequency is low and results appear highly variable (also see troubleshooting problem 3). In the original manuscript corresponding to this protocol, we provide a detailed description of MADM GT and TG cassettes for all 19 mouse autosomes including primer sequences for genotyping (Contreras et al., 2021).

We provide an example in Figure 2 using the candidate gene *Ndel1*, which is located on chromosome 11, and thus needs to be recombined to MADM chromosome 11. MADM-11 cassettes are ∼3 cM distal to the centromere and *Ndel1* is located ∼42 cM distal to the centromere, or ∼39 cM away from the MADM cassettes. Thus, there is ∼39% probability that meiotic recombination will occur in the recombinant generating mating. However, since only half of the progeny will be of the desired genotype (TG,mutant), while the other half will be wild-type (+,+), animals with the appropriate genotype will be generated at a rate of ∼20% (39% × ½) (Figure 2A).

6. The larger the genetic distance between the MADM cassette and the genomic locus of your gene of interest, the higher the recombination probability. The scale of the mating for generating recombinants using meiotic recombination needs to be adjusted according to the recombination probability. In other words, the probability of meiotic recombination determines the amount of matings you require to set up in order to successfully obtain mice where MADM cassettes and genes of interest are genetically linked. The higher the recombination probability, the less breeding cages will be needed to obtain recombinants.

***Note:*** All information provided in this section is specific to the example gene *Ndel1* in order to provide numerical calculation support to the reader. The numbers and probabilities for your specific experiments require adjustments based on your genes of interest and the required MADM chromosomes.

7. The MADM targeting constructs were introduced into C57BL/6N embryonic stem cells and injected into blastocysts isolated from BALB/cRj females. The offspring were bred with C57BL/6NRj females. Further backcrossing of offspring positive for MADM cassettes was performed with CD1 mice to obtain MADM stock lines of mixed CD1-C57BL/6J genetic background. Consider variances in litter size or phenotype based on mouse background, particularly when you are usually employing pure inbred mice for your experiments.

## Key resources table

**Table:** 

REAGENT or RESOURCE	SOURCE	IDENTIFIER
**Chemicals, peptides, and recombinant proteins**		

PFA	Sigma-Aldrich	Cat# 441244
10**×** PBS	Thermo Fisher Scientific	Cat# 70011
Triton X 100	Sigma-Aldrich	Cat# T878
Tween 20	Sigma-Aldrich	Cat# P9416
0.2M Tris HCL pH 8.5	CARL ROTH	Cat# 9090
NaH_2_PO_4_·2H_2_O	Sigma-Aldrich	Cat# 567549
Na_2_HPO_4_	Sigma-Aldrich	Cat# S3264
Glycerol	Sigma-Aldrich	Cat# G5516
Citric Acid	CARL ROTH	Cat# X863.1
NaOH	CARL ROTH	Cat# T198.1
Heat-inactivated Horse Serum (HS)	Thermo Fisher Scientific	Cat#26050088
Avertin (2,2,2 triboromoethanol)	Sigma-Aldrich	Cat# T48402
t-amylalcohol (2-methyl-2-butanol)	Sigma-Aldrich	Cat# 240486
Ethanol	Honeywell	Cat# 24194
Sucrose	Sigma-Aldrich	Cat# S8501
O.C.T. Tissue Tek	Sakura	Cat# 4583
Mowiol 4-88	CARL ROTH	Cat# 0713
DABCO	CARL ROTH	Cat# 0718
DAPI	Molecular Probes	Cat# D1306

**Antibodies**		

Chicken anti-GFP antibody, 1:400	Aves	RRID:AB_10000240
Goat anti-tdT antibody, 1:400	SICGEN ANTIBODIES	RRID:AB_2333092
rabbit anti-PhosphoH3 Ser10, 1:800	CellSignaling Technology	RRID:AB_1549592
Donkey anti-chicken-FITC secondary antibody, 1:500	Invitrogen	RRID:AB_923386
Donkey anti-goat Alexa568 secondary antibody, 1:1000	Molecular Probes	RRID:AB_2534104
Donkey anti-rabbit Alexa647 secondary antibody, 1:1000	Molecular Probes	RRID:AB_2762835

**Experimental models: Organisms/strains**		

Mouse: MADM-7-GT	The Jackson Laboratory	RRID:IMSR_JAX:021457
Mouse: MADM-7-TG	The Jackson Laboratory	RRID:IMSR_JAX:021458
Mouse: MADM-11-GT	The Jackson Laboratory	RRID:IMSR_JAX:013749
Mouse: MADM-11-TG	The Jackson Laboratory	RRID:IMSR_JAX:013751
Mouse: MADM-18-GT	(Contreras et al., 2021)	N/A
Mouse: MADM-18-TG	(Contreras et al., 2021)	N/A
Mouse: *Emx1*-Cre	The Jackson Laboratory	RRID:IMSR_JAX:005628
Mouse: *Emx1*-CreER	The Jackson Laboratory	RRID:IMSR_JAX:027784
Mouse: *Nestin*-Cre	(Petersen et al., 2002)	N/A
Mouse: *Nestin*-CreER	(Imayoshi et al., 2006)	N/A
Mouse: *Hprt*-Cre	The Jackson Laboratory	RRID:IMSR_JAX:004302
Mouse: *Apc*-flox	The Jackson Laboratory	RRID:IMSR_JAX:009045
Mouse: *TrkC*	(Tessarollo et al., 1997)	N/A
Mouse: *Ndel1*	The Jackson Laboratory	RRID:IMSR_JAX:026958
Mouse: *Lgl1-*flox	(Klezovitch et al., 2004)	N/A

**Software and algorithms**		

ZEN blue	ZEISS	http://www.zeiss.com/microscopy/en_us/products/microscope-software/zen.html#introduction
Photoshop	Adobe	adobe.com/products/photoshop
GraphPad Prism	GraphPad	https://www.graphpad.com/scientific-software/prism/

**Other**		

Histosette (embedding cassette for tissue fixation)	Sakura	Cat# 4170-01
Embedding molds for peripheral organs	Sakura	Cat# 4557
Embedding molds for coronal brain sections	Polysciences, Inc..	Cat# 18986-1
Embedding molds for sagittal brain sections	Polysciences, Inc.	Cat# 18646A-1
6 well plates	TPP	Cat# 92406
24 well plates	TPP	Cat# 92424
Flask Filters 500mL	TPP	Cat# 99505
Microfuge Tubes 1.5 mL	Thermo Fisher Scientific	Cat# AM12450
50 mL Centrifuge Tubes	Sarstedt, Inc.	Cat# 62.547.254
15 mL Centrifuge Tubes	Sarstedt, Inc.	Cat# 62.554.502
Syringe 60mL	Kendall	Cat# 560125
Syringe 10 mL Omnifix	B.Braun	Cat# 4617100V
Needle 20G Sterican	B.Braun	Cat# 4657705
0.2micron filter	Nalgene	Cat# 194-2520
Peristaltic Pump	Watson-Marlow	Cat# 323 S/D
Hydrophobic Pen	DAKO	Cat# S2002
Petri dish	Thermo Fisher Scientific	Cat# NC9565080
Slide Moisture Chamber black	Newcomer Supply	Cat# 68432A
Superfrost Glass Slides	Thermo Fisher Scientific	Cat# J1800AMNZ
Cover Slips 24 × 50 mm	VWR	631–0147
Dissection Tools	Freudenberg FST	Various, depending on the specific needs of the experiment
Fine Brush Size 1	Ted Pella, Inc.	Cat# 11859
LSM 800 Confocal	ZEISS	N/A
Cryostat Cryostar NX70	Thermo Fisher Scientific	N/A

## Materials and equipment

### Essential buffers and anesthesia for tissue harvesting (prepare 1 day prior to the experiment)

**Timing: 60 min**

**Table:** 1M PB buffer (pH 7.4)

Reagent	Final concentration	Amount
2M NaH_2_PO_4_^.^ 2H_2_O (monobasic sodium phosphate)	1M	47.5 mL
2M Na_2_HPO_4_ (dibasic sodium phosphate)	1M	202.5 mL
ddH_2_O	n/a	250 mL
**Total**	**1M**	**500 mL**

Keep at 22°C–25°C.

**Table:** 2N NaOH

Reagent	Final concentration	Amount
NaOH	2N	40 g
ddH_2_O	n/a	500 mL
**Total**	**2N**	**500 mL**

Keep at 22°C–25°C.

**Table:** 4% PFA (in 0.1M PB buffer)—always prepare fresh PFA

Reagent	Final concentration	Amount
Paraformaldehyde (PFA)	4%	40 g
2N NaOH	0.01N	4 mL
1M PB buffer	0.1M	100 mL
ddH_2_O	n/a	850 mL
**Total**	**4%**	**1,000 mL**

Details for PFA preparation: (1) Dissolve 40 g of PFA in 750 mL distilled water. (2) Add 4 mL 2N NaOH. (3) Place on a stirring heating plate at 55°C until all PFA has dissolved. (4) Add 100 mL 1M PB buffer. (5) Fill up the volume to 1000 mL with distilled water. (6) Filter through a flask filter to avoid residual undissolved PFA particles. (7) Store at 4°C for up to 1–2 days.

**Table:** Anesthesia stock solution (100%)

Reagent	Final concentration	Amount
Avertin	100%	7 g
t-amylalcohol	n/a	7 mL
**Total**	**100%**	**7 mL**

Details for anesthesia preparation: (1) Vortex until the solution appears homogeneous. (2) Wrap in tin foil to protect from light and store at 22°C–25°C for up to 6 months.

**Table:** Anesthesia working solution (2.5%)

Reagent	Final concentration	Amount
100% anesthesia stock solution	2N	0.875 mL
1**×** PBS	1**×**	34.125 mL
**Total**	**2.5%**	**35 mL**

Details for anesthesia preparation: (1) Vortex until the solution appears homogeneous. (2) Filter the solution into a fresh tube using a 60 mL syringe and a 0.2micron filter. (3) Store at 4°C for up to one month.

**Table:** 1**×** PBS:

Reagent	Final concentration	Amount
10**×** PBS	1**×**	100 mL
ddH_2_O	n/a	900 mL
**Total**	**1×**	**1,000 mL**

Keep at 22°C–25°C.

**Table:** Sucrose

Reagent	Final concentration	Amount
Sucrose	30%	300 g
1**×** PBS	1**×**	Add up to 1,000 mL
**Total**	**30%**	**1,000 mL**

We recommend to filter the sucrose solution through a 500 mL flask filter to avoid contamination with microorganisms. Store at 4°C.

### Prepare perfusion equipment (on the day of experiment)

**Timing: 15 min**

• Clean all dissection tools (2 curved forceps, large and small scissors) with 70% ethanol.
• Prepare a perfusion pad and whipping tissues.
• Prepare a peristaltic pump or two 10 mL syringes and needles for perfusion.
• Prepare 6-well plates filled with 4% PFA to collect brain tissues.
• Prepare histosettes and a beaker filled with 4% PFA to collect peripheral organs.

### Prepare essential buffers and reagents for histological stainings (on the day of the experiment)

**Table:** Cryoprotectant

Reagent	Final concentration	Amount
Glycerol	60%	300 mL
1M PB buffer	0.1M	50 mL
ddH_2_O	n/a	150 mL
**Total**	**60%**	**500 mL**

Store at 4°C.

**Table:** PBS with 0.5% Tween (PBS-T)

Reagent	Final concentration	Amount
Tween20	0.5%	5 mL
1**×** PBS	1**×**	995 mL
**Total**	**0.5%**	**1000 mL**

Add 5 mL Tween20 to 1,000 mL 1**×** PBS and mix well. Store at 22°C–25°C.

**Table:** Citrate buffer pH 6.0

Reagent	Final concentration	Amount
Citric acid anhydrous (192M)	1M	1.92 g
Tween20	0.05%	0.5 mL
ddH_2_O	n/a	1,000 mL
**Total**	**1M**	**1,000 mL**

Details for citrate buffer preparation: (1) Dissolve 1.92 g citric acid anhydrous (192M) in 1000 mL distilled water. (2) Adjust to pH 6.0 using NaOH. (3) Add 0.5 mL of Tween20. (4) Store at 4°C.

**Table:** Triton-X-100 working solution

Reagent	Final concentration	Amount
Triton-X-100	20%	2 mL
ddH_2_O	n/a	8 mL
**Total**	**20%**	**10 mL**

Store it in a 15 mL Falcon tube at 22°C–25°C.

**Table:** Blocking buffer

Reagent	Final concentration	Amount
Horse Serum	10%	1 mL
Triton-X-100 working solution	0.5%	0.25 mL
1**×** PBS	1**×**	8.75 mL
**Total**	**n/a**	**10 mL**

Store it in a 15 mL Falcon tube at 4°C.

**Table:** Staining buffer

Reagent	Final concentration	Amount
Horse Serum	5%	0.5 mL
Triton-X-100 working solution	0.5%	0.25 mL
1**×** PBS	1**×**	9.25 mL
**Total**	**n/a**	**10 mL**

Store it in a 15 mL Falcon tube at 4°C.

***Note:*** You can prepare 1,000 ml of Blocking and Staining Buffer and aliquot in 50 mL or 15 mL tubes, which can be stored at −20°C until further usage. Once thawed, keep at 4°C and use up within 1 week.

**Table:** DAPI stock solution

Reagent	Final concentration	Amount
1 vial DAPI	5 mg/mL	10 mg
ddH_2_O	n/a	2 mL
**Total**	**5 mg/mL**	**2 mL**

Aliquot in 1.5 mL tubes and store at −20°C.

**Table:** DAPI working solution

Reagent	Final concentration	Amount
DAPI stock solution	300nM	1 μL
1**×** PBS	1**×**	20 mL
**Total**	**300nM**	**20 mL**

Store at 2°C–6°C up to one week, protected from light.

**Table:** Embedding medium Mowiol-DABCO

Reagent	Final concentration	Amount
Glycerol	n/a	6 g
Mowiol	n/a	4 g
ddH_2_O	n/a	6 mL
0.2M Tris-HCl (pH 8.5)	0.1	12 mL
DABCO	n/a	25 mg
**Total**	**n/a**	**25 mL**

Details for Mowiol-DABCO preparation: (1) Use a balance to weight 6.0 g Glycerol and 2.4 g Mowiol. Given the high viscosity of Glycerol, it is more accurate to measure its weight than its volume. (2) Mix both reagents and dissolve for 1 h on a stirring plate. (3) Add 6 mL distilled water and mix for another hour. (4) Add 12 mL 0.2M Tris-HCl (pH 8.5) and incubate for 2 h at 50°C on a stirring heating plate. (5) It is likely that not all Mowiol is going to dissolve. Thus, you can add an additional centrifugation step to remove undissolved Mowiol from the preparation by centrifuging for 15 min at 5,000 g and keeping the supernatant. (6) Add 25 mg DABCO and dissolve so that you have a clear solution. (7) Aliquot in 1.5 mL tubes and store at −20°C until further usage.

## Step-by-step method details

### Breeding of experimental MADM mice for the generation of genetic mosaic tissue

**Timing: 4–7 months**

In order to generate genetic mosaic mice, you can either chose a breeding strategy maximizing the efficiency of experimental MADM animal output (here called “strategy 1”) or a breeding strategy maximizing the speed of obtaining experimental MADM animals (here called “strategy 2”) (Figures 2B and 2C). We provide an estimated amount of breeding mice required to obtain a realistic number of experimental MADM animals (Figures 2B and 2C). We provide step-by-step breeding guidance in the following section of this protocol (also see troubleshooting problem 1):

1. For both breeding strategies, perform Breeding step 1 for *gene-X* of interest: In order to link the mutation of *gene-X* to the MADM TG cassette, cross *MADM*^*TG/TG*^ animals with a mouse line mutant for *gene-X* (Figures 2B(i) and 2C(i)). The resulting F1 generation will be trans-heterozygous for the MADM TG cassette and the mutation of interest (genotype will be *MADM*^*TG/+*^*; gene-X*^*+/-*^).
2. For both breeding strategies, perform Breeding step 1 for the Cre driver (Figures 2B(iii) and 2C(ii)): cross *MADM*^*GT/GT*^ animals with a Cre driver of choice. The resulting F1 generation will be double heterozygous for the MADM GT cassette and the Cre driver specific for the cell type of interest (*MADM*^*GT/+*^*; Cre*^*+/-*^). Perform this step in parallel to the above step.

Allow F1 generation mice to grow up to 8 weeks of age. For breeding strategy 2, proceed to step 6. For breeding strategy 1, continue with steps 3, 4 and 5.

3. For strategy 1, perform Breeding step 2 for the mutant by crossing *MADM*^*TG/+*^*;gene-X*^*+/-*^ mice with *MADM*^*TG/TG*^ animals in order to generate F2 offspring homozygous for the MADM TG cassette and meiotically linked *gene-X* (*MADM*^*TG/TG,gene-X*^) (Figure 2B(ii)).
4. For strategy 1, perform Breeding step 2 for the Cre driver by intercrossing *MADM*^*GT/+*^*; Cre*^*+/-*^ mice with *MADM*^*GT/+*^*; Cre*^*+/-*^ animals in order to generate F2 offspring homozygous for the MADM GT cassette and the Cre driver. The resulting genotype will be *MADM*^*GT/GT*^*; Cre*^*+/+*^ (Figure 2B(iv)). Perform this step parallel to step 3.

Allow F2 generation mice to grow up to 8 weeks of age.

5. For the generation of experimental MADM animals according to strategy 1, cross *MADM*^*TG/TG,gene-X*^ mice with *MADM*^*GT/GT*^*; Cre*^+/+^ animals in order to generate experimental offspring with genetic mosaic tissue with green mutant, red wild-type and yellow heterozygous cells (Figure 2B(v)).
  The genotype of the experimental MADM animals is *MADM*^*GT/TG,gene-X*^*; Cre*^*+/-*^ (Figure 2B(v)).
  The probability to obtain experimental MADM animals from this breeding is 50%. The probability for control-MADM animals is also 50%, i.e., animals with MADM-labelling but without mutation. With an estimated litter size of 10 pups per litter, this means that you require 1 breeding cage to obtain a total of 5 experimental MADM mice (Figure 2B(vi)).
  a. Check for the vaginal plug to monitor successful mating and to expedite the experimental planning.
  b. Male and female offspring can be used as experimental animals.
  c. Perform experiment at developmental stage(s) of choice.

6. For the generation of experimental MADM animals according to strategy 2, cross trans-heterozygous *MADM*^*TG/+*^*;gene-X*^*+/-*^mice directly with double heterozygous *MADM*^*GT/+*^*; Cre*^*+/-*^ animals in order to generate experimental MADM offspring with genetic mosaic tissue with green mutant, red wild-type and yellow heterozygous cells (Figure 2C(iii)).
  The genotype of the experimental MADM animals is *MADM*^*GT/TG,gene-X*^*; Cre*^*+/-*^ (Figure 2C(iii)).
  The probability to obtain experimental MADM animals from this breeding is ∼5%. With an estimated litter size of 10 pups per litter, you require ∼10 breeding cages and ∼100 pups to obtain a total of 5 experimental MADM mice (Figure 2C(iv)).
  a. Check for the vaginal plug to monitor successful mating and to expedite the experimental planning.
  b. Male and female offspring can be used as experimental animals.
  c. Perform experiment at developmental stage(s) of choice.
    ***Note:*** In the following examples described in this protocol, all tissue was analyzed at specific postnatal stages. However, experimental animals can also be used at embryonic stages, P0, and any postnatal age depending on your experimental needs.
    In order to provide a broad overview of applications suitable for histological analysis using MADM in this protocol, we made use of a number of different Cre drivers: (1) constitutively active *Emx1*-Cre and TM-inducible *Emx1-*CreER that primarily targets radial glia progenitors in the dorsal forebrain, which give rise to excitatory projection neurons and glial cells in the cerebral cortex (Gorski et al., 2002; Kessaris et al., 2006). (2) We include an example using constitutively active *Nestin*-Cre and inducible *Nestin-*CreER (Petersen et al., 2002; Imayoshi et al., 2006), which is a marker of stem cells across the nervous system and therefore results in MADM labeling in all neural cell types including Purkinje cells; (3) and make use of constitutively active *Hprt*-Cre (Tang et al., 2002) which shows ubiquitous spatiotemporal expression. The *Hprt*-Cre driver was utilized to investigate peripheral organs, in particular the intestine.
    MADM can also be applied for experiments involving flow cytometry. For details and a step-by-step protocol on the experimental workflow specific for flow cytometry experiments, we refer to (Laukoter et al., 2020a).
    Specific considerations when breeding experimental MADM animals are provided in the next two sections of the protocol:

### Generation of experimental MADM mice for postnatal histological analysis in brain

**Timing: 3 weeks–12 months**

**Figure 3:**
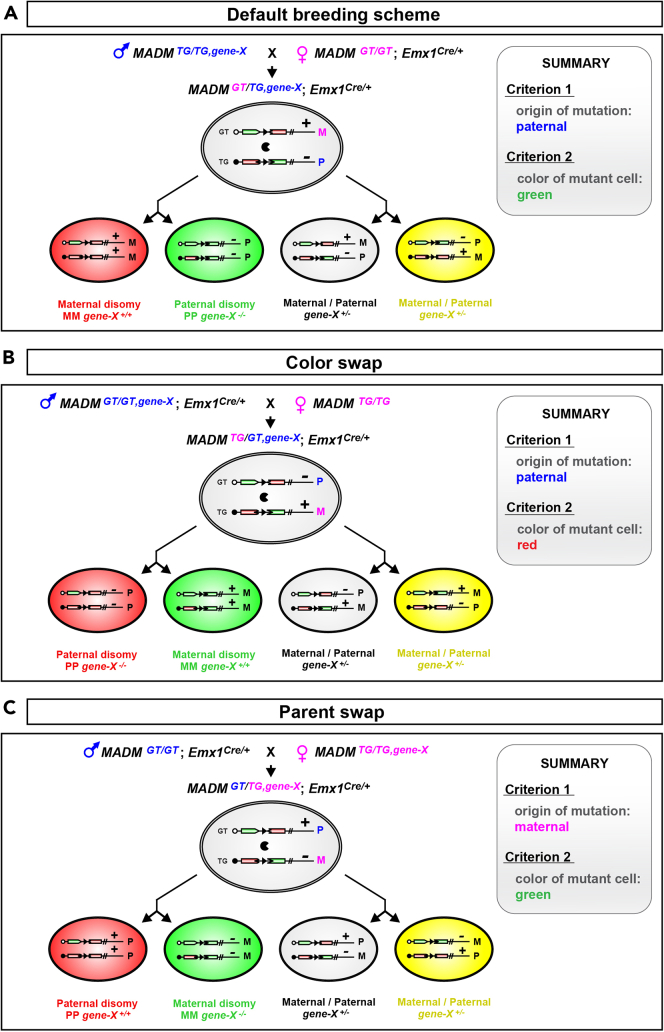
Breeding schemes for default setup, color swap, and parent swap (A) Schematic showing the default breeding scheme using males where *gene-X* is meiotically linked to the MADM TG cassette, while females transmit the GT cassette and the Cre driver. Resulting offspring will contain red wild-type cells with uniparental maternal chromosome disomy (matUPD, MM), while green mutant cells will harbor uniparental paternal chromosome disomy (patUDP, PP). (B) Schematic of the breeding scheme for a color swap experiment using females homozygous for the MADM TG cassette, while males transmit the Cre driver plus *gene-X* meiotically linked to the GT cassette. Resulting offspring will have green wild-type cells with matUPD (MM), while red mutant cells will harbor patUPD (PP). (C) Schematic of the breeding scheme for a parent swap experiment using females where *gene-X* is meiotically linked to the MADM TG cassette, while males transmit the GT cassette and the Cre driver. Resulting offspring will contain red wild-type cells with patUPD (PP), while green mutant cells will harbor matUPD (MM). Parts of the figure were reproduced and adapted with permission from (Beattie et al., 2017).

7. For population analysis, cross *MADM*^*GT/GT*^*; Emx1*^*Cre/Cre*^ or *MADM*^*GT/GT*^*; Nestin-Cre*^*+*/*+*^ females with *MADM*^*TG/TG;gene-X*^ males in order to generate offspring with genetic mosaic brain tissue (Figure 2B, bottom panel) (also see troubleshooting problem 4).
  For clonal analysis, cross *MADM*^*GT/GT*^*; Emx1*^*CreER/CreER*^ or *MADM*^*GT/GT*^*; Nestin-CreER*^*+/+*^ females with *MADM*^*TG/TG;gene-X*^ males in order to generate offspring with genetic mosaic brain tissue following TM injection at a time point of choice (Figure 2B, bottom panel) (also see troubleshooting problem 5).
  For simplicity, the above mentioned distinct Cre/ER drivers will be abbreviated as “Cre” throughout step 7.
  a. Check for the vaginal plug to monitor successful mating and to facilitate the experimental planning.
  b. Males and females of the resulting F1 generation can be used as experimental animals (genotype will be *MADM*^*GT/TG,gene-X*^*; Cre*^*+/-*^*).*
    ***Note:*** Upon crossing *MADM*^*GT/GT*^*; Cre*^+/+^ females with *MADM*^*TG/TG,gene-X*^ males to generate *MADM*^*GT/TG,gene-X*^*; Cre*^*+/-*^ mosaic animals, green cells harboring a homozygous mutation in *gene-X* will show uniparental paternal chromosome disomy (patUPD, PP), while red wild-type cells will show uniparental maternal chromosome disomy (matUPD, MM) (Figure 3A). We refer to this breeding scheme as ‘default breeding scheme.’
    ***Note:*** In order to reverse the fluorescent colors to control for a potential bias of the fluorescent reporter (color swap), couple the mutation to the MADM GT cassette and use *MADM*^*TG/TG*^ females and *MADM*^*GT/GT,gene-X*^*; Cre*^*+/+*^ males (Laukoter et al., 2020a). Here, red cells harboring a homozygous mutation in *gene-X* will show patUPD (PP), while green wild-type cells will show matUPD (MM) (Figure 3B). For efficient conduction of this control experiment, we recommend to perform the color swap in the context of population analysis using a constitutively active Cre driver and not a TM-inducible CreER.
    ***Note:*** In order to reverse the parents to control for a potential genomic imprinting bias of the parental origin of the mutation (parent swap), use *MADM*^*TG/TG,gene-X*^ females and *MADM*^*GT/GT*^*; Cre*^*+/+*^ males (Laukoter et al., 2020a) to generate *MADM*^*GT/TG,gene-X*^*; Cre*^*+/-*^ mosaic animals. Here, green cells harboring a homozygous mutation in *gene-X* will show matUPD (MM), while red wild-type cells will show patUPD (PP) (Figure 3C). Given that the generation of stock lines for your experiments will yield *MADM*^*TG/TG,gene-X*^ females and *MADM*^*GT/GT*^*; Cre*^*+/+*^ males in any case, use the opportunity of having these mice available and perform the parent swap in parallel to your ‘default breeding’ setup. For efficient conduction of this control experiment, we recommend to perform it in the context of population analysis using a constitutively active Cre driver and not a TM-inducible CreER.
  c. Perform experiment at your desired postnatal time point, for example when generation of cell types is completed and the cells have reached a fully developed, mature state (in our examples shown in this protocol we chose time points between P1 and P21).

### Generation of experimental MADM mice for postnatal histological analysis of genetic mosaic tissue in peripheral organs

**Timing: 3 weeks–12 months**

8. Cross *MADM*^*GT/GT*^*; Hprt*^*Cre*/+^ females with *MADM*^*TG/TG,gene-X*^ males in order to generate offspring with genetic mosaic tissues.
  a. Check for the vaginal plug to monitor successful mating and to facilitate the experimental planning.
  b. Males and females of the resulting F1 generation can be used as experimental animals (genotype will be *MADM*^*GT/TG,gene-X*^*; Hprt*^*Cre*/+^).
    **CRITICAL:** The *Hprt*-Cre transgene is located on the X chromosome, with one of the two X chromosomes undergoing random inactivation in females. Thus, Cre activity and MADM labelling are highly variable in *MADM*^*GT/TG,gene-X*^*; Hprt*^*Cre/*+^ females. We therefore recommend to primarily use males for your analysis in order to obtain reproducible results.
    ***Note:*** Upon crossing *MADM*^*GT/GT*^*; Hprt*^*Cre*/+^ females with *MADM*^*TG/TG,gene-X*^ males to generate *MADM*^*GT/TG,gene-X*^*; Hprt*^*Cre*/+^ mosaic animals, green cells harboring a homozygous mutation in *gene-X* will show patUPD, while red wild-type cells will show matUPD.
    ***Note:*** If desired, perform color swap and parent swap (Laukoter et al., 2020a) as described in the notes for step 7 of the protocol.
  c. Perform the experiment at the desired postnatal time point, to e.g., analyze cellular phenotypes arising during development in mature tissue and/or trace disease phenotypes when a disease phenotype is evident (up to several months of age).

### Mouse perfusion and dissection for collection of MADM-labelled tissue for histological analysis

**Timing: 12–16 h**

This section of the protocol documents the perfusion and dissection of postnatal mice for organ removal and further downstream histological analysis.

**CRITICAL:** 4% PFA should be ice-cold and protected from light.

9. Arrange all solutions and tools required for the tissue collection at the dissection area
  a. Anesthesia
  b. 1**×** PBS
  c. 4% PFA
  d. Peristaltic pump or perfusion syringes
  e. Dissection tools
  f. 6-well plates

**CRITICAL:** Make sure no air bubbles are trapped in the tubes of the peristaltic pump or within the syringe containing the perfusion buffers. Bubbles may destroy the tissue during the perfusion process. Run the tubes of the pump with PBS prior to starting the perfusion procedure to make sure all air is removed.

10. Anesthetize the animal by intraperitoneal injection of anesthesia working solution at a volume of 0.16–0.24 mL/10 g mouse (equivalent to 400–600 mg/kg).

**CRITICAL:** Mice require deep anesthesia before any further procedure can be applied. Test the efficiency of anesthesia by pinching a paw with forceps. Only when the mouse does not display any movement after pinching, you can proceed with the dissection and perfusion.

11. Place the anesthetized animal in supine position on a perfusion tray and disinfect fur with 70% ethanol.
12. Below the rib cage, make a medial incision with scissors and surgical forceps through the skin and the underlying muscle layer (Figure 4A). Continue to cut laterally.

**Figure 4:**
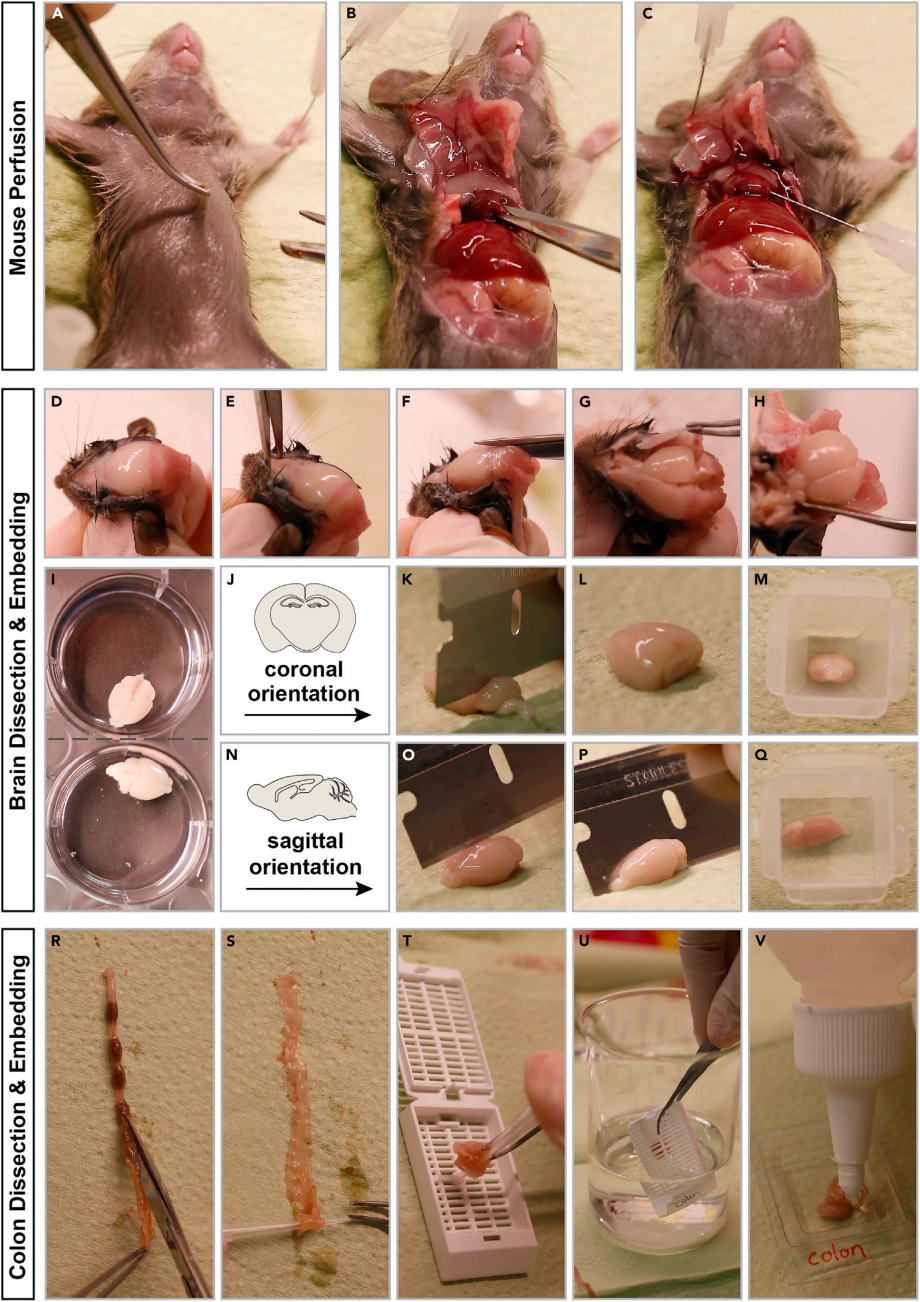
Tissue harvesting for histological analysis of experimental MADM mice (A–C) Mouse dissection and initiation of perfusion. (D–Q) Mouse dissection to isolate and embed brain. (R–V) Isolation and embedding of colon.
13. Lift the tip of the sternum and snip the diaphragm. Then trim the rib cage to expose the heart.
14. Using small iris scissors make an incision to the posterior end of the right atrium for the blood to drain during the following perfusion procedure (Figure 4B).
15. Insert a needle attached to a peristaltic pump or a syringe filled with 1**×** PBS into the lower left ventricle (Figure 4C).
16. Perfuse with PBS until the liver turns pale (requires approximately 5–10 mL of PBS).
17. Once complete, switch the perfusion solution to 4% PFA.
  ***Note:*** Perfusion requirements need to be adjusted according to the age of the animals. (a) For the perfusion of pups: perfuse manually using 10 mL syringes. Prepare 2 distinct 10 mL syringes: one filled with 1**×** PBS and another filled with 4% PFA. Once perfusion with PBS is completed, replace PBS-containing syringe plus needle with fresh needle and syringe containing PFA through the same puncture hole in the heart. (b) For the perfusion of juvenile and adult mice, use a peristaltic pump. Place one end of the tube of the peristaltic pump into a flask or Falcon tube filled with PBS. After perfusion with PBS (speed: 5 mL at 2–4 mL/min in juvenile animals; 10 mL at 4–6 mL/min for adult mice), switch to 4% PFA.
  **CRITICAL:** briefly stop the peristaltic pump and exchange the PBS solution with ice-cold 4% PFA. This is critical to avoid bubbles in the pump tubing. Resume perfusion with PFA at the same speed.

18. Remove mouse from perfusion pad and dissect the organ of interest. If organ of interest is brain, continue to follow the protocol. If organ of interest is a peripheral organ, proceed to step 27.
19. Dissection of brain:
  a. Decapitate mouse.
  b. Remove the skin over the skull (Figure 4D).
  c. Cut the skull at the naso-frontal suture (in front of the olfactory bulbs) (Figure 4E).
  d. Then cut along the midline (Figure 4F), followed by a cut along the parietal-interparietal sutures.
  e. Lift the skull to the side to expose the brain (Figure 4G).
  f. Place curved forceps or a thin spatula underneath the brain and remove it from the skull (Figure 4H).

**CRITICAL:** Avoid mechanical damage of brain tissue during the dissection to ensure you obtain intact tissue for high quality analysis.

20. Transfer the brain to a well filled with ice-cold 4%PFA in a 6-well plate (Figure 4I).
21. Incubate at 4°C for minimum 10 h.
22. Next day, wash 3**×** with 1**×** PBS for 5 min each.
23. Transfer brain to a 15 mL Falcon tube filled with 10 mL of 30% Sucrose.
24. Incubate at 4°C until the brain has sunk to the bottom of the Falcon tube.
25. Remove brain from sucrose and gently remove residual sugar from the surface of the brain using lint-free tissues.
26. Depending on the cell type of interest and the needs of the experiment, embed brain in coronal or sagittal orientation:
  a. For coronal orientation (Figure 4J), place brain ventral-side down on a piece of lint-free tissue and remove the cerebellum using a razor blade (Figures 4K and 4L). Place brain with the caudal side down in an embedding mold (Figure 4M), fill mold and cover tissue with O.C.T. Tissue tek and freeze. Once frozen, store it at −80°C.
  b. For sagittal orientation (Figure 4N), place brain ventral-side down on a piece of lint-free tissue and cut it in two halves along the midline using a razor blade (Figures 4O and 4P). Place brain with the medial side down in an embedding mold (Figure 4Q), fill mold and cover tissue with O.C.T. Tissue tek and freeze. Once frozen, store it at −80°C.
    **Pause point:** You can keep the frozen tissue at −80°C until further usage.

27. Dissection of peripheral organ (this protocol is using the colon as an example):
  a. Remove colon from the mouse and place it on lint-free tissue.
  b. Cut open longitudinally (Figure 4R).
  c. Remove faeces.
  d. Make colon into a Swiss roll by using a pipet tip to roll it up (Figure 4S).
  e. Transfer colon into a histosette (Figure 4T) for further handling.
28. Incubate in a beaker filled with 4% PFA for 4 h at 22°C–25°C (Figure 4U).
29. Wash 3**×** with 1**×** PBS for 5 min each.
30. Incubate in 30% Sucrose at 4°C for minimum 10 h.
31. Remove histosette with colon from sucrose, take out colon and gently remove residual sugar from the surface of the organ by using lint-free tissues.
32. Transfer colon to an embedding mold, fill mold and cover tissue with O.C.T. Tissue Tek and freeze (Figure 4V). Once frozen, store it at −80°C.

**Pause point:** You can keep the frozen tissue at −80°C until further usage.

**CRITICAL:** Optimal tissue freezing during embedding is achieved when embedding molds are placed on dry ice. Control the tissue during the freezing process from time to time and avoid loss of position within the mold.

### Tissue sectioning for histological analysis

**Timing: 30 min–3 h**

This section of the protocol lists details on sectioning MADM-labelled tissue for confocal microscopy.

33. Remove tissue from −80°C and transfer to a cryostat cooled to −20°C.
34. Let tissue adapt to temperature change for 20 min.
35. Remove tissue block from the embedding mold.
36. Attach the tissue block to the specimen disk in the cryostat by applying a drop of OCT to the disk and placing the tissue block directly into the OCT. Allow to freeze for 1–2 min.
37. Insert disk with attached tissue block in the sectioning holder by placing the tissue in a correct orientation.

***Note:*** For brain sections, insert sectioning disc with cortex facing towards the top. For peripheral organ sections, insert sectioning disc with organ of choice in an orientation that guarantees good sectioning quality for your required analysis.

38. Start cutting by trimming the tissue block in 45 μm thick sections until the region of interest is reached.
39. Slice the tissue in 30 μm thick sections. Either collect the slices in individual wells of a 24 well plate (= floating sections) or mount them directly onto frosted glass slides.

**Pause point:** Floating sections can be kept in PBS at 4°C for up to 48 h. When longer storage is required, exchange the PBS with cryoprotectant and store at −20°C.

**Pause point:** Directly mounted sections can be stored in a slide box at −20°C until further usage.

### Tissue mounting

**Timing: 15 min–1 h**

This part of the protocol can be skipped if the sliced tissue is directly mounted onto frosted glass slides in the course of the sectioning process. In that case, immediately proceed to step 45 of the protocol. If you prepared floating sections, continue with step 40.

40. Fill a Petri dish with PBS-T.
41. Place a frosted glass slide at the edge of the petri dish so that is covered up nearly to the label with PBS-T.
42. Transfer all of your desired sections into the dish filled with PBS-T.
43. Use a small paint brush to maneuver the sections onto the slide and arrange them side by side.
44. Once all sections are placed onto the slide (for brain usually ∼12−16 sections/slide), horizontally transfer the slide to a dark slide chamber and let the sections dry completely (∼15 min) to make them adhere to the glass. Either proceed to the next steps of the staining procedure or pause the protocol at this point.

**Pause point:** Store the mounted slides at 4°C for 10–16 h and continue with the protocol on the next day.

### Staining

**Timing: 3–16 h**

This section of the protocol explains the staining procedure for MADM-labelled cells together with an additional, cell-type specific marker staining. All steps are performed at 22°C–25°C unless otherwise stated. Please refer to troubleshooting problem 2 when observing variable signals.

45. If your sections were floating sections and have been freshly mounted and dried, immediately proceed to step 46 of the protocol. If your sections were already mounted during sectioning and stored at −20°C, take the slides from the freezer and let them dry for 15 min.
46. Encircle staining area with a hydrophobic pen.
47. Rehydrate and wash sections 3**×** 5 min with 1**×** PBS to remove residual PBS-T.
48. Meanwhile, heat up an oven to 85°C. Take the opportunity to put the antigen retrieval buffer into the oven to have the solution pre-heated. Our most commonly used antigen retrieval solution is Citrate Buffer pH 6.0.
49. Incubate your sections with 1 mL pre-heated citrate buffer pH 6.0 for 25 min at 85°C in a dark and closed staining chamber (make sure it is closed. If it is open, buffer will evaporate and samples will dry out).
50. Remove the staining chamber and let chill on your bench for 15 min. Open it on one corner a tiny bit so the samples can cool down more efficiently (don’t open too much, otherwise the buffer will evaporate and samples will dry out).
51. Wash 3**×** 5 min with 1**×** PBS.
52. Incubate with 500 μL Blocking Solution for 1 h.
53. Place humidified tissues into the staining chamber to prevent your samples from dehydration during the next steps of the protocol.
54. Dilute primary antibodies in Staining Solution, add 250 μL antibody mix for one sample and incubate 8–12 h at 4°C.
55. Wash 3**×** 5 min with 1**×** PBS.
56. Dilute secondary antibodies in Staining Solution, add 250 μL antibody mix for one sample and incubate for 2 h.
57. Wash 3**×** 5 min with 1**×** PBS.
58. Add 1 mL DAPI working solution for 15 min.
59. Wash 1**×** 10 min in PBS
60. Embed the slides with Mowiol and let dry at 22°C–25°C for 30 min.

**Pause point:** Keep the slides at 4°C until imaging. Image quality is highest upon timely acquisition of images following the staining procedure. We recommend to image the sections within 14 days after completion of step 60.

***Note:*** Instead of mounting the floating sections onto glass slides prior to the staining procedure, the whole staining protocol can be performed on floating sections in a 24well plate. Buffers are removed manually using a pasteur pipet or a P1000. We recommend the following volumes when performing stainings in 24well plates:

**Table:** 

Buffer volumes per well for staining of floating cryosections	
Reagent	Volume (μl)
PBS	1,000
Antigen retrieval buffer	500
Blocking Buffer	500
Staining Buffer (including antibody mix)	250
DAPI working solution	500

Here, sections should be mounted after the final washing step following the secondary antibody staining. Mounting process is equivalent to steps 40 to 44 of the protocol. Once mounting and section drying is completed, you can proceed with DAPI staining and Mowiol embedding (steps 58 to 60 of the protocol).

**Table:** Antibodies and their dilutions for MADM staining

Antibody	Species	Dilution
Anti-GFP	Chicken	1:400
Anti-mCherry	Goat	1:400
Anti-chicken FITC	Donkey	1:500
Anti-goat Alexa568	Donkey	1:1000

**Table:** Antibodies and their dilutions for additional marker staining, here an example for staining performed in the colon (Figure 6)

Antibody	Species	Dilution
Anti-PhosphoH3	Rabbit	1:800
Anti-rabbit Alexa647	Donkey	1:1000

**Figure 5:**
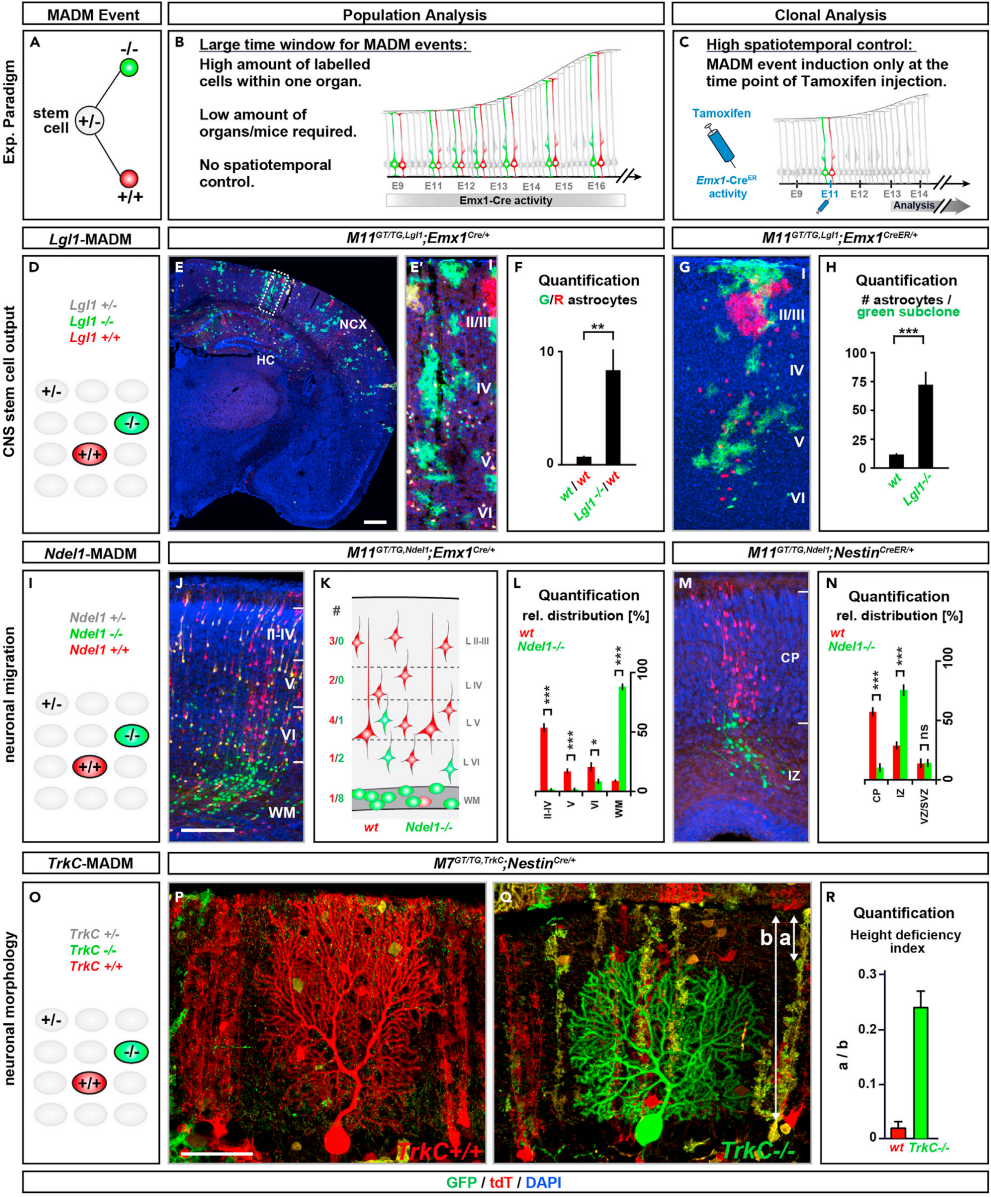
Examples and analysis of genetic mosaic MADM animals with mosaicism in the brain (A–C) Schematic of G2-X MADM events resulting in (A) genetic mosaic tissue using (B) constitutively active Cre driver for population analysis and (C) tamoxifen-inducible CreER driver for clonal analysis. (D–H) Analysis of central nervous system (CNS) stem cell output in *Lgl1*-MADM mice using *Lgl1* mutant allele (Klezovitch et al., 2004). (D) schematic of *Lgl1*-MADM cortical tissue; (E and F) population analysis of *Lgl1* using *Emx1*-Cre driver (Gorski et al., 2002) with (E) overview of a representative brain section at P21, (E′) insert showing the cortex, and (F) quantification of the G/R ratio of astrocytes from wild-type mice and *Lgl1*-MADM mice; (G and H) clonal analysis of *Lgl1* using *Emx1*-CreER driver (Kessaris et al., 2006) with (G) reconstruction of a representative clone at P21 and (H) quantification of the number of green astrocytes in green subclones for wild-type and *Lgl1* mutant clones. Scale bars: (E) 500 μm, (E′) 60 μm, (G) 70 μm). Values represent mean ± SEM. ns, nonsignificant, ∗∗∗p < 0.001. Parts of the figure and its legend were adapted and reused from (Beattie et al., 2017) with permission. (I–N) Analysis of cortical projection neuron migration in *Ndel1*-MADM mice using *Ndel1* knock-out allele (Sasaki et al., 2005). (I) schematic of *Ndel1*-MADM cortical tissue; (J–L) population analysis of *Ndel1* using *Emx1*-Cre driver (Gorski et al., 2002) with (J) overview of a representative image of the cortex section at P1, (K) schematic indicating partitioning of the cortex into layers and layer-specific quantification of red and green neurons, and (L) quantification of the relative distribution of red wild-type and green *Ndel1* mutant cells in the respective layers; (M and N) clonal analysis of *Ndel1* using *Nestin*-CreER driver [line 1 in (Imayoshi et al., 2006)] with (M) reconstruction of a representative clone at E18.5 and (N) quantification of the relative distribution of red wild-type and green *Ndel1* mutant cells in the respective layers. Scale bars: (J) 150 μm, (M) 100 μm. Values represent mean ± SEM. ns, nonsignificant; ∗p < 0.05 and ∗∗∗p < 0.001. Parts of the figure and its legend were adapted and reused from (Hippenmeyer et al., 2010) with permission. (O–R) Analysis of Purkinje cell morphology in *TrkC*-MADM mice using *TrkC* knock-out allele (Tessarollo et al., 1997) and *Nestin*-Cre driver (Petersen et al., 2002). (O) schematic of *TrkC*-MADM neuronal tissue; (P and Q) representative high magnification images of (P) red wild-type and (Q) green *TrkC* mutant Purkinje cells in the cerebellum of P21 *TrkC*-MADM mice, (R) quantification of the height deficiency index of red wild-type and green *TrkC* mutant cells within the Molecular layer of the cerebellum. Scale bars: (P and Q) 50 μm. Presented morphology values are means ± SEM. ∗∗∗ p < 0.001, one-way analysis of variance (ANOVA) with Tukey’s multiple comparisons test. Parts of the figure and its legend were adapted and reused from (Joo et al., 2014) with permission.

### Microscopy and image processing

**Timing: 1–12 h**

In this section of the protocol, we describe the microscopy settings and subsequently required confocal image processing for further downstream analysis.

61. For the analysis of MADM-labeled tissue we recommend to use an inverted confocal microscope, in our case Zeiss LSM800 with corresponding image acquisition software ZEN blue.
62. Select the correct laser lines and filters. For MADM tissue with additional marker staining, select excitation: 405 nm (for DAPI), 488 nm (for FITC / GFP), 561 nm (for Alexa568 / tdT) and 640 nm (for Alexa647).
63. Set to pinhole to 1 airy unit for optimal imaging quality.
64. Select the functions “Z-stack” and “Tiles” from the acquisition dashboard.
65. Select objective, scanning speed, resolution and averaging appropriate to the needs of your experiments.
66. Adjust laser intensity and gain for each channel.
67. Identify your area of interest and set position and imaging tiles to cover the area of interest. Adjust the z-stack so that all MADM-labeled cells in the tissue are captured.
68. Assign the center position of your Z-stack to the tiles and the positions and click “Start” to initiate image acquisition.
69. Save the image in .czi file format.

***Note:*** For tissue overview images and cell number quantification, select a 10**×** objective and use a scanning speed pixel dwell value of 1.52–2.06 μs (values 7–8 in the ZEN blue image acquisition software) with resolution 1024 **×** 1024 and no averaging. For high-quality images and morphological analysis, use a high magnification objective (e.g. 63**×**) and use a scanning speed pixel dwell value of maximum 2.06 μs (up to value 7 in the ZEN blue image acquisition software) with resolution 1024 **×** 1024 and 2**×** or 4**×** averaging.

**Pause point:** The protocol can be stopped at this point. Image processing and analysis can be performed at any time after image acquisition.

70. Once image acquisition is completed, use the image processing dashboard in ZEN blue and perform maximum intensity projection using the “Orthogonal Projection” function.
71. Following projection, perform “Stitching” function by fusing tiles.
72. Save the projected and stitched image as a new .czi file.
73. Export the projected and stitched .czi file to TIFF format. Export DAPI / GFP / tdT / Alexa647 channels individually for subsequent image analysis.

## Expected outcomes

Compare your readout of green and red cells (such as cell number, cell position, morphological parameters, presence of tumors) in genetic mosaic MADM animals. Since the genotypes of red cells (wild-type) and green cells (mutant) are different, any deviation from a comparable green-to-red (G/R) read-out indicates a cell-autonomous function of *gene-X* in your cell type of interest.

In order to study stem cell output by counting cell numbers, determine the G/R cell ratio of your cell type of interest (Figures 5F and 5H): If G/R ratio were above 1, it indicates a proliferation increase of green mutant stem cells, whereas a G/R ratio below 1 indicates cell loss either through diminished stem cell proliferation or apoptosis of either stem cells or their descendants. Thus, *gene-X* has a cell-autonomous function in governing overall stem cell output.

To study cell migration, measure cell position and the number of red wild-type and green mutant cells in certain positions (Figures 5L and 5N): A deviation of numbers of green cells vs. red cells in particular bins is indicative of a cell migration alteration. A large absence of green mutant neurons from cortical layers and their accumulation in the white-matter demonstrates a cell-autonomous function of *gene-X* in neuronal migration. Based on the sparse endogenous labeling with GFP and tdT, MADM is also well suited to study cell migration in live imaging assays and 4D tracing with high resolution (Riccio et al., 2016; Hippenmeyer et al., 2010).

Determine morphological parameters, such as the height index (Figure 5R): calculate the height index by the formula a/b (a = the distance from the pial surface to the top of the arbor of a Purkinje cell; b= the molecular layer span) and compare the values for red wild-type and green mutant cells side-by-side. If green mutant cells display higher values for height parameters than red wild-type cells, this result indicates a decreased height of the analyzed cells. As a conclusion, *gene-X* exerts a cell-autonomous function in cellular morphology.

**Figure 6:**
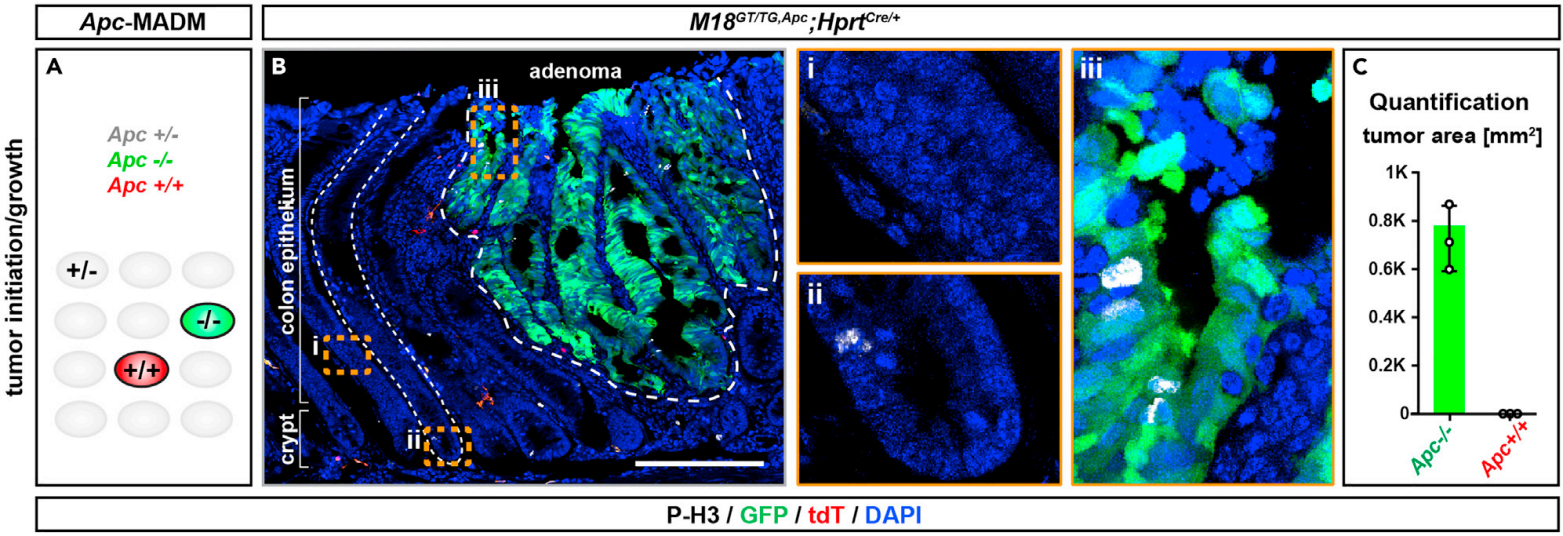
Example and analysis of genetic mosaic MADM mice with mosaicism in peripheral organs (A–C) MADM analysis of *Apc* tumor suppressor gene in the colon using *Apc*-flox allele (Cheung et al., 2010) and *Hprt*-Cre driver (Tang et al., 2002). (A) schematic of *Apc*-MADM tissue; (B) representative image of an adenoma arising from green *Apc* mutant stem cells in the colon of a 3month old *Apc*-MADM mouse. Inserts (i) and (ii) show unlabeled healthy colon epithelium, where proliferating cells (as indicated by M-Phase marker staining with P-H3 antibody) are only located in the crypt bottom, but not above the crypt region or along the differentiated epithelium. In contrast insert (iii) highlights a subepithelial region within the adenoma displaying proliferating cells. (C) Quantification of the tumor area originating from red wild-type and green *Apc* mutant cells. Scale bars: (B) 200 μm, (i, ii, iii) 40 μm. Values represent means ± standard deviation.

In order to study a disease model such as tumorigenesis (Figure 6C), compare the numbers and area of red and green tumors in your tissue of choice. An increased number of green tumors and tumor area indicates a cell-autonomous function of *gene-X* in stem cell proliferation and tumor initiation, whereas a decreased number indicates a cell-autonomous disadvantage in tumor growth.

## Quantification and statistical analysis

**Timing: several hours or days**

Once a stem cell undergoes a G2-X MADM event, one green labelled mutant and one red labelled wild-type daughter cell is generated in an unlabeled environment (Figure 5A). Such sparsely labelled cells can be subjected to a variety of downstream analyses, such as lineage tracing, cell number quantification, positional analysis or morphological analysis (Figure 5).

These distinct types of downstream analysis can be performed for both, population and clonal analysis. While population analysis results in sparse MADM-labeling with no spatiotemporal information (Figure 5B) clonal analysis employing a TM-inducible CreER driver can provide high spatiotemporal resolution (Figure 5C).

The following steps of the protocol provide a short description of the distinct types of histological analysis enabled by the MADM-induced genetic mosaics (also see troubleshooting problem 3):

1. For the determination of stem cell output, quantify the number of red and green cells in your acquired images using the counting tool in Photoshop. To do this, load all individual channel images into one image stack to keep channels separate. Use the counting tool to manually quantify the number of distinct cell types derived from red wild-type and green mutant stem cells. Afterwards, calculate and plot the green-to-red (G/R) cell ratio. (Figures 5D–5H).

***Alternatives:*** if photoshop is not accessible, alternative freely accessible software such as Fiji can be used for the quantification of green and red cells. In Fiji, use the tool ‘multi-points’ for quantification.

2. For the assessment of cellular migration, partition the tissue into defined layers or bins. Next, quantify the number of individual red wild-type and green mutant cells within these layers or bins by using the ‘counting’ tool in photoshop. Plot the cell number per bin for red and green cells side-by-side and compare their distribution to determine if mutant cells are found in a different position than wild-type cells (Figures 5I–5N).
3. In order to determine cellular morphology, import your .czi image into a software appropriate for morphological analysis, such as Imaris and use the filament tracer function. Determine parameters such as cell volume or complexity using Sholl analysis. Compare the values for red wild-type and green mutant cells to assess if the mutation causes a morphology phenotype (Figures 5O–5R). For assessment of cell height, define specific parameters, such as the distance from the pial surface to the top of the arbor of a Purkinje cell (parameter a) and the molecular layer span (parameter b) (Figure 5Q). Such parameters can be measured with the ‘ruler’ tool using ZEN software on the .czi file of your image or in photoshop or Fiji using other image formats such as .tif.
4. For disease modeling such as the investigation of tumor initiation and growth, quantify the number of individual tumors using the counting tool in photoshop or determine the size of the tumor area by delineation of the tumor margin in ZEN blue or in photoshop (Figure 6).

## Limitations

The genome-wide library of MADM mice allows to employ MADM to study the vast majority, nearly genome-wide (>96%), of autosomal genes in the mouse genome. Mouse autosomes have a telocentric conformation and we thus inserted the MADM cassettes as close as possible to the centromere to maximize the number of genes located distally to the MADM cassette insertion site. In order to be meiotically linked to the corresponding MADM chromosome, the gene of interest needs to be located distally to the MADM cassette. The MADM principle relies on meiotic recombination to genetically link a mutation to the MADM cassette. However, too close proximity of the gene of interest to the MADM cassette might make a recombination event difficult or impossible. In such cases, other techniques offering sparse induction of a mutation of choice could be used, such as MADR (Kim et al., 2019), Dual ifgMosaic (Pontes-Quero et al., 2017) or BATTLE (Kohara et al., 2020).

The library of MADM mice currently does not include strains with MADM cassettes inserted on the sex chromosomes, thus while the vast majority (>96%) of genes can be studied, the genes located on the X or Y chromosome are not yet covered by the MADM technique.

## Troubleshooting

### Problem 1

Low number of experimental mice (breeding of experimental MADM mice – steps 1–6).

### Potential solution 1

Make sure the genetic background of your mice does not exhibit negative effects on their mating performance. Increase the number of breeding cages to gain a good number of F1 or F2 mice to start with.

Furthermore, control whether you have correctly followed the provided breeding strategies. Wrongly genotyped or selected parental mice can result in strongly decreased efficiency in the generation of experimental animals.

### Problem 2

Weak or variable MADM signal without staining for GFP and tdT (Staining – steps 45–60).

### Potential solution 2

Endogenous fluorescence intensity of GFP and tdT are pH sensitive. Make sure all of your buffers have the correct pH.

Additionally, perform staining for GFP and tdT using the antibodies indicated above.

### Problem 3

Highly variable results in MADM lines with low recombination frequency (Quantification and Data Visualization – steps 74–77).

### Potential solution 3

Analysis of all 19 MADM lines showed that different MADM chromosomes harbor distinct recombination frequencies [sparse, intermediate or dense, (Contreras et al., 2021)]. The required number of experimental animals and the number of tissue sections used for analysis need to be scaled up when working with a MADM chromosome that gives sparse labeling. We recommend using the entire amount of tissue sections available for one animal to avoid introduction of an analysis bias when only using a few sections from a low recombination line.

### Problem 4

Low breeding performance (Generation of experimental MADM mice for histological analysis, steps 7–8).

### Potential solution 4

Discuss with your veterinarian how to optimize the mating conditions (e.g., by providing special food or housing conditions).

### Problem 5

High abortion rate upon Tamoxifen injection for clonal analysis (Generation of experimental MADM mice for histological analysis, step 7).

### Potential solution 5

Titrate the concentration of Tamoxifen according to your experimental needs to find an optimal balance between survival and clone numbers. Additionally, recover pups by caesarian sections and perform fostering to increase the survival rate of experimental animals.

## Resource availability

### Lead contact

Further information and requests for resources and reagents should be directed to and will be fulfilled by the lead contact Simon Hippenmeyer (simon.hippenmeyer@ist.ac.at).

### Materials availability

This study did not generate new unique reagents.

## Data Availability

No custom code is necessary to perform this protocol.
